# HDAC4 drives ferroptosis and fibrosis by inhibiting Foxo3a-GPX4 axis during AKI–CKD progression

**DOI:** 10.21203/rs.3.rs-7809768/v1

**Published:** 2025-10-27

**Authors:** Shougang Zhuang, Fengchen Shen, xinyu du, Liyuan Yao, Chao Yu, Yanjin Wang, jianjun yu, zhipeng yan, Yuzhen Zhang, Na Liu

**Affiliations:** Department of Medicine; Shanghai East Hospital; Shanghai East Hospital; Shanghai East Hospital; Shanghai East Hospital, Tongji University School of Medicine; Shanghai East Hospital; Shanghai East Hospital; Shanghai East Hospital; Shanghai East Hospital, Tongji University School of Medicine; Shanghai East Hospital, Tongji University School of Medicine

## Abstract

Histone deacetylase 4 (HDAC4) modifies both histone and non-histone proteins, but its role in the transition from acute kidney injury (AKI) to chronic kidney disease (CKD) remains unclear. Here, we investigated the function and mechanism of HDAC4 in ischemia–reperfusion (IR)–induced AKI–CKD progression using Tasquinimod, a highly selective HDAC4 inhibitor, and conditional tubular HDAC4 knockout mice. We found that HDAC4 expression was persistently upregulated after IR and was associated with sustained ferroptosis. Both pharmacological inhibition and tubular deletion of HDAC4 suppressed ferroptosis, alleviated tubular injury, and reduced fibrosis. Mechanistically, HDAC4 promoted ferroptosis by regulating the nucleocytoplasmic shuttling of Foxo3a: it enhanced Foxo3a phosphorylation, bound Foxo3a in the cytoplasm, and induced its deacetylation, collectively sequestering Foxo3a in the cytoplasm and reducing GPX4 transcription. Inhibition or deletion of HDAC4 restored Foxo3a nuclear localization, upregulated GPX4, and decreased lipid peroxidation. These findings identify HDAC4 as a key mediator linking IR injury to ferroptosis and fibrotic progression, suggesting that targeting the HDAC4–Foxo3a axis may provide a novel therapeutic strategy to prevent the AKI–CKD transition.

## Introduction

Acute kidney injury (AKI) is a critical clinical condition characterized by a rapid decline in renal function. Its global prevalence is increasing, making it a major threat to human health[1, 2]. When AKI persists for more than seven days without recovery of renal function or resolution of structural damage, it often progresses to chronic kidney disease (CKD)[2, 3]. Common causes of AKI include ischemia-reperfusion (IR) injury, rhabdomyolysis, and nephrotoxic drugs, which mainly damage renal tubules and impair kidney function[2, 3, 4]. Despite advances in renal replacement therapy, there are still no effective drugs to halt the progression of AKI to CKD.

IR injury remains a major cause of AKI, primarily due to reduced renal blood flow and tubular obstruction[5, 6]. Following AKI, tubular epithelial cells (TECs) undergo apoptosis and necrosis, while surviving TECs attempt to repair through proliferation and redifferentiation[7]. However, incomplete epithelial repair, coupled with persistent inflammation, promotes fibrosis, ultimately leading to glomerulosclerosis, tubulointerstitial extracellular matrix (ECM) deposition, and capillary rarefaction[8, 9]. A key pathogenic driver of IR injury is oxidative stress, which induces mitochondrial dysfunction, plasma membrane damage, and lipid peroxidation[10, 11].

Ferroptosis is a newly recognized form of regulated cell death driven by iron-dependent lipid peroxidation, first described in 2012[12], that is driven by iron-dependent lipid peroxidation. Unlike apoptosis or necrosis, ferroptosis is characterized by shrunken mitochondria with condensed cristae and outer membrane rupture, without overt plasma membrane rupture[12, 13]. Ferroptosis is triggered by glutathione peroxidase 4 (GPX4) inactivation or by free iron ions through the Fenton reaction[12]. GPX4 is critical for detoxifying lipid peroxides and thus prevents ferroptosis by maintaining redox homeostasis[14]. Another key regulator, Acyl-CoA synthetase long-chain family member 4 (ACSL4), promotes ferroptosis by facilitating lipid biosynthesis and increasing lipid peroxidation substrates[15]. Ferroptosis has been linked to various diseases, including acute liver injury, Alzheimer’s disease, and cancer[16, 17, 18], underscoring the importance of clarifying its cellular pathways.

Recent evidence suggests that the transcription factor Foxo3a may regulate ferroptosis. Foxo3a is a member of the Forkhead box (Fox) family, defined by a conserved DNA-binding domain with a winged-helix structure[19]. As a central mediator of cellular responses to oxidative stress, DNA damage, and cell cycle arrest, Foxo3a is implicated in longevity and tumor suppression[20, 21]. Its function depends largely on its subcellular localization, which is dynamically regulated by post-translational modifications[22, 23]. When phosphorylated, Foxo3a is exported to the cytoplasm and inactivated; when acetylated, it relocates to the nucleus to activate transcription[24, 25, 26, 27]. However, how Foxo3a modulates ferroptosis through its phosphorylation or acetylation status remains poorly understood.

Studies have shown that protein acetylation plays a critical role in the progression from AKI to CKD. For example, dynamic changes in the acetylation of both histone and non-histone proteins has been observed during renal IR injury, underscoring the importance of epigenetic regulation in kidney damage[28]. Histone acetylation typically occurs on specific lysine residues at the amino termini of histones and is dynamically regulated by histone acetyltransferases (HATs) and histone deacetylases (HDACs)[29]. Among these, HDAC4—a class IIa HDAC with a conserved catalytic domain—can shuttle between the cytoplasm and nucleus. HDAC4 has been implicated in multiple kidney diseases, including lipopolysaccharide-induced AKI, unilateral ureteral obstruction–induced CKD, and diabetic nephropathy[30, 31, 32]. However, its role in IR-induced AKI and the subsequent transition to CKD remains poorly understood.

In this study, we investigated HDAC4 expression and function in IR-induced AKI and CKD using the specific HDAC4 inhibitor tasquinimod and conditional HDAC4 knockout mice. Our findings demonstrate that HDAC4 promotes ferroptosis and fibrosis by suppressing GPX4 through Foxo3a signaling during the AKI–CKD transition. These results suggest that targeting HDAC4 may offer a novel therapeutic strategy to prevent or delay the progression of AKI to CKD, ultimately improving outcomes for patients with ischemic kidney injury.

## Results

### HDAC4 expression is increased in IR-induced acute and chronic kidney disease

**Figure 1:**
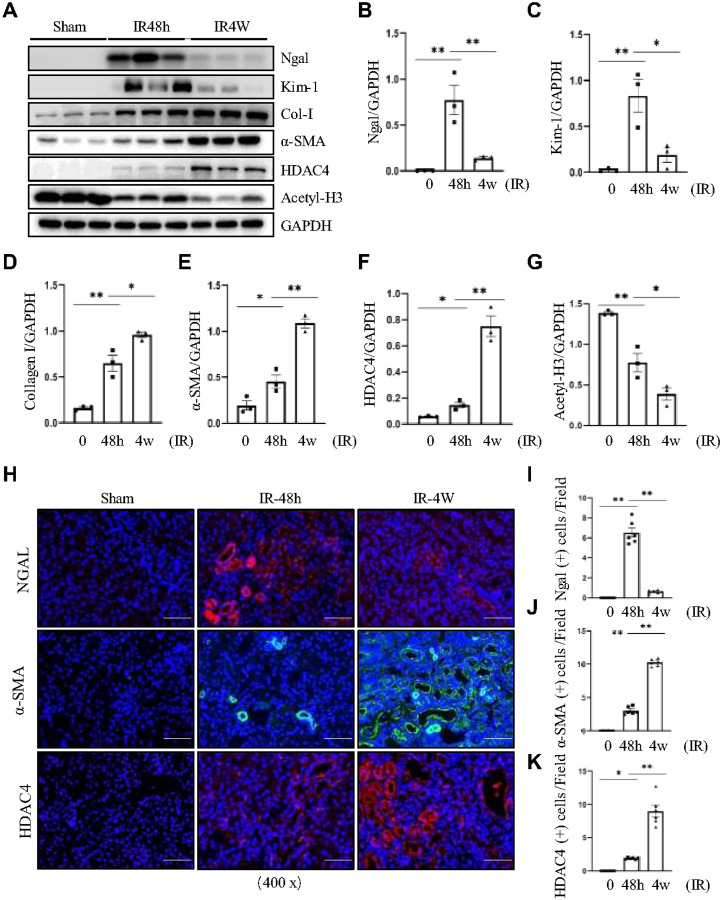
HDAC4 expression is increased in IR-induced acute and chronic kidney disease. (A)The kidney tissue lysates were subjected to immunoblot analysis with antibodies against Ngal, Kim-1, collagen I, α-SMA, HDAC4, Acetyl-Histone 3 and GAPDH. Expression levels of Ngal (B), Kim-1 (C), collagen I (D), α-SMA (E), HDAC4 (F) and Acetyl-Histone 3 (G) were quantified by densitometry and normalized using GAPDH. Values are the means ± SEM of 3 samples. *P < 0.05; **P < 0.01. (H) Micrographs of Ngal, α-SMA and HDAC4 immunofluorescence staining (magnification ×400, Scale bar= 50μm). Ngal (I), α-SMA (J) and HDAC4 (K) staining shows quantitative data. Data are presented as mean± SEM (n=6 per group). *P < 0.05; **P < 0.01.

We first examined changes in HDAC4 expression during the AKI–CKD transition by establishing a unilateral renal IR model with contralateral nephrectomy for AKI and a unilateral renal IR model with contralateral kidney preservation for CKD. As shown in Fig. 1A–C, renal tubular injury was evident in both IR-induced AKI and CKD mice, with injury markers Ngal and Kim-1 elevated to varying degrees, more markedly in IR-induced AKI. Consistent with progressive fibrosis, expression of fibronectin (FN), collagen I (Col I), and α-SMA was significantly increased in IR-induced CKD, and HDAC4 expression was higher in CKD than in AKI (Fig. 1A, D–G). Immunofluorescence staining confirmed that tubular injury was more severe in IR-induced AKI (Fig. 1H, I). Due to incomplete repair in the acute phase, HDAC4 and α-SMA expression increased progressively with advancing renal fibrosis (Fig. 1H, J, K).

In addition, we validated these findings using a folic acid (FA)-induced AKI–CKD model. In this model, Ngal and Kim-1 expression was elevated in FA-induced AKI compared with FA-induced CKD. Col I and α-SMA levels exhibited a marked increase in the FA-induced 4-week model (Figure S1A–E). Consistent with findings in the IR model, HDAC4 expression increased at 48 hours after folic acid (FA) administration and was further elevated at 4 weeks. These results indicate that HDAC4 activation begins during acute kidney injury and is sustained and amplified during fibrotic progression (Figure S1A, F).

### Conditional knockout or pharmacologic inhibition of HDAC4 attenuates IR-induced AKI

**Figure 2:**
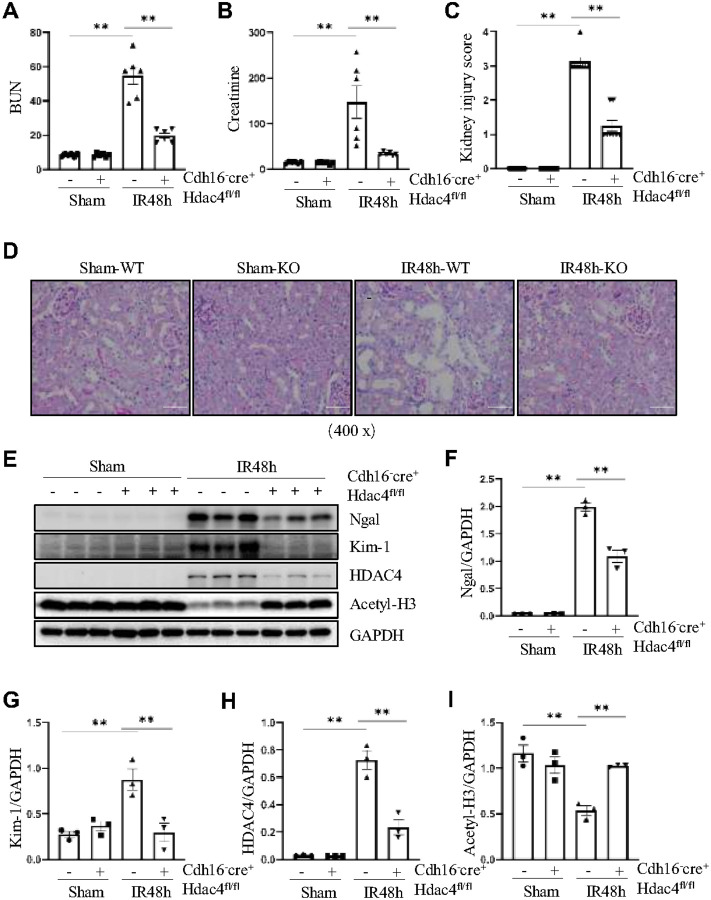
Pharmacologic inhibition or conditional knockout of HDAC4 attenuates IR-induced AKI. Cdh16-Cre^−^: HDAC4^fl/fl^ (HDAC4-WT) and Cdh16-Cre^+^: HDAC4^fl/fl^ (HDAC4-KO) mice were sacrificed 48 hours after sham operation or IR. (A, B) Blood urea nitrogen (BUN) levels and serum creatinine were measured using a colorimetric method in each group of mice. (C, D) Periodic acid-Schiff (PAS) staining revealed kidney structure damage in mice from each group. Data are presented as mean ± SEM (n = 6 per group). Scale bar = 50 μm. Tubule injury scores of renal tissue were assessed in each group. (E) The kidney tissue lysates were subjected to immunoblot analysis with antibodies against Ngal, Kim-1, HDAC4, acetyl-histone 3, and GAPDH. Expression levels of Ngal (F), Kim-1 (G), HDAC4 (H) and acetyl-histone 3 (I) were quantified by densitometry and normalized using GAPDH. Values are presented as mean ± SEM of three samples. *p < 0.05; **p < 0.01.

To determine the role of HDAC4 in renal IR injury, we first investigated the effect of conditional knockout HDAC4 on IR-induced Ngal and Kim-1, two hallmarks of renal tubular injury. Renal tubule–specific HDAC4 knockout mice were generated by crossing HDAC4fl/fl mice with Cdh16-Cre mice, yielding Cdh16-Cre+:HDAC4fl/fl mice (HDAC4-KO) and Cdh16-Cre-:HDAC4fl/fl mice (HDAC4-WT) littermates (Figure S2A). Successful HDAC4 depletion and elevated histone acetylation were confirmed by reduced HDAC4 and increased acetyl-H3 expression in KO kidneys (Fig. 2E, H, I). At 48 hours post-IR, HDAC4-KO mice exhibited significantly improved renal function compared with WT controls, as shown by lower serum creatinine and BUN levels (Fig. 2A, B). PAS staining demonstrated that IR caused severe tubular injury, including tubular dilation, which was markedly alleviated in HDAC4-KO mice, indicating better preservation of renal architecture (Fig. 2C, D). Western blot results further showed that HDAC4 knockout reduced the expression of renal injury markers Kim-1 and Ngal, confirming the protective effect (Fig. 2E–G).

To investigate the pharmacological effect of HDAC4 inhibition on the IR-induced AKI, we treated the mouse model of AKI with/without tasquinimod, a selective HDAC4 inhibitor, immediately after reperfusion (Figure S2B). Inhibition of HDAC4 activity by tasquinimod significantly reduced serum creatinine and blood urea nitrogen levels and alleviated renal tubular damage in IR-injured mice (Figure S3A-3D). Western blot analysis confirmed that renal IR injury upregulated HDAC4, Ngal, and Kim-1 expression, which was reversed by tasquinimod treatment, accompanied by increased H3K9 acetylation (Figure S3E-I).

Collectively, these findings indicate that HDAC4 contributes to IR-induced AKI and that pharmacologic inhibition or genetic knockdown of HDAC4 effectively ameliorates renal injury.

### Pharmacologic inhibition or conditional knockdown of HDAC4 attenuates IR-induced renal fibrosis

**Figure 3:**
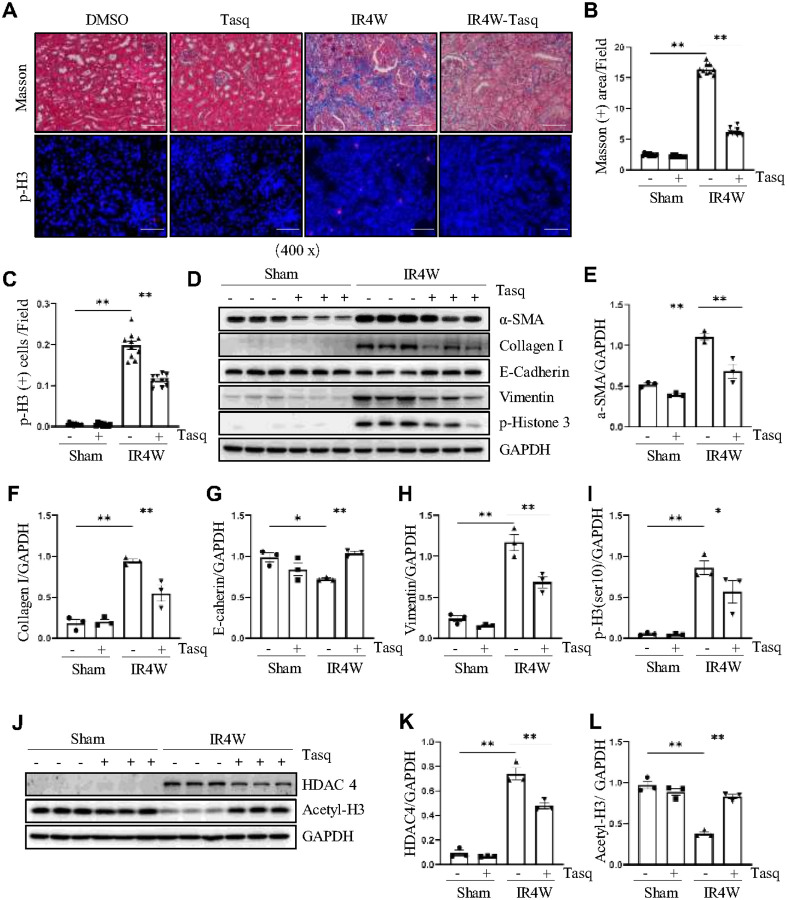
Pharmacologic inhibition or conditional knockdown of HDAC4 attenuates IR-induced renal fibrosis. (A) Micrographs of Masson’s trichrome staining, magnification ×400, Scale bar = 50 μm. Micrographs of p-H3 immunofluorescence staining (magnification ×400, Scale bar = 50 μm) of the kidney tissues collected at 48 h after sham and IR injury with or without tasquinimod administration in C57/black mice. (B) Graph shows the positive area of the Masson-positive tubulointerstitial fibrosis (blue) from 10 random fields of kidney samples from three mice. (C) p-H3 staining shows quantitative data. Data are presented as mean ± SEM (n = 10 per group). Scale bar =50 μm. (D) The kidney tissue lysates were subjected to immunoblot analysis with antibodies against α-SMA, Collagen I, E-cadherin, Vimentin, p-H3 and GAPDH. Expression levels of α-SMA (E), Collagen I (F), E-cadherin (G), Vimentin (H), and p-H3 (I) were quantified by densitometry and normalized using GAPDH. (J) The kidney tissue lysates were subjected to immunoblot analysis with antibodies against HDAC4, acetyl-histone 3 and GAPDH. Expression levels of HDAC4 (K) and acetyl-histone 3 (L) were quantified by densitometry and normalized using GAPDH. Values are presented as mean ± SEM of three samples. *p < 0.05; **p < 0.01.

We also used both the selective HDAC4 inhibitor tasquinimod and renal tubule–specific HDAC4 knockout mice to evaluate the role of HDAC4 in IR-induced renal fibrosis. First, tasquinimod was administered to mice with IR-induced renal fibrosis to assess its effects on renal pathology and fibrosis marker expression (Figure S2C). Masson’s trichrome staining revealed substantial renal interstitial fibrosis at 4 weeks post-IR, which was significantly reduced following tasquinimod administration two days after IR (Fig. 3A, B). In addition, inhibition of HDAC4 alleviated cell cycle arrest in renal fibrosis, as evidenced by p-H3 immunofluorescence and western blot analysis (Fig. 3A–D, I). Compared with untreated IR-induced CKD mice, tasquinimod treatment reduced the expression of α-SMA, collagen I, vimentin, but preserved the expression of E-cadherin (Fig. 3D–H). Notably, administration of tasquinimod effectively reduced HDAC4 expression and increased histone acetylation levels, confirming its inhibitory efficacy (Fig. 3J–L).

**Figure 4:**
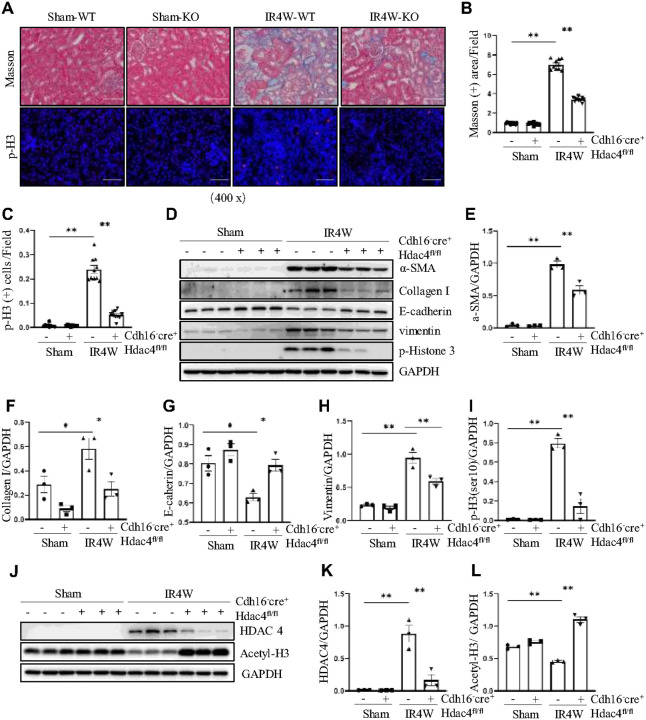
Pharmacologic inhibition or conditional knockdown of HDAC4 attenuates IR-induced renal fibrosis. Cdh16-Cre^−^: HDAC4^fl/fl^ (HDAC4-WT) and Cdh16-Cre^+^: HDAC4^fl/fl^ (HDAC4-KO) mice were sacrificed 4 weeks after sham operation or IR. (A) Micrographs of Masson’s trichrome staining, magnification ×400, Scale bar = 50 μm. Micrographs of p-H3 immunofluorescence staining (magnification ×400, Scale bar = 50 μm) of the kidney tissues collected at 4 weeks after sham and IR injury. (B) Graph shows the positive area of the Masson-positive tubulointerstitial fibrosis (blue) from 10 random fields of kidney samples from three mice. (C) p-H3 staining shows quantitative data. Data are presented as mean ± SEM (n = 10 per group). Scale bar =50 μm. (D) The kidney tissue lysates were subjected to immunoblot analysis with antibodies against α-SMA, Collagen I, E-cadherin, Vimentin, p-H3 and GAPDH. Expression levels of αSMA (E), Collagen I (F), E-cadherin (G), Vimentin (H), and p-H3 (I) were quantified by densitometry and normalized using GAPDH. (J) The kidney tissue lysates were subjected to immunoblot analysis with antibodies against HDAC4, acetyl-histone 3 and GAPDH. Expression levels of HDAC4 (K) and acetyl-histone 3 (L) were quantified by densitometry and normalized using GAPDH. Values are presented as mean ± SEM of three samples. *p < 0.05; **p < 0.01.

Consistent results were observed in the HDAC4 conditional knockout model. HDAC4 knockdown markedly attenuated renal interstitial fibrosis, with renal histopathology showing less interstitial fibrosis and reduced cell cycle arrest in HDAC4-KO mice compared with HDAC4-WT mice at 4 weeks post-IR (Fig. 4A–C). IR-induced upregulation of α-SMA, collagen I, E-cadherin, vimentin, and p-Histone H3 in the chronic fibrosis model was significantly suppressed by HDAC4 knockdown (Fig. 4D–I). Western blot confirmed efficient HDAC4 knockdown by showing decreased HDAC4 and increased ace-H3 expression (Fig. 4J–L).

Furthermore, consistent findings were obtained in vitro using mTECs stimulated with TGF-β1. TGF-β1 treatment increased HDAC4 expression, reduced acetyl-histone H3K9 levels, and markedly upregulated the fibrosis markers α-SMA and collagen I. HDAC4 knockdown by siRNA restored histone acetylation and significantly attenuated fibrosis in mTECs. (Figure S4A–E).

Together, these results demonstrate that pharmacological inhibition or targeted knockdown of HDAC4 effectively reverses IR-induced renal fibrosis, partial EMT and fibroblast activation.

### HDAC4 inhibition or deficiency protects against IR-induced AKI by reducing ferroptosis

**Figure 5:**
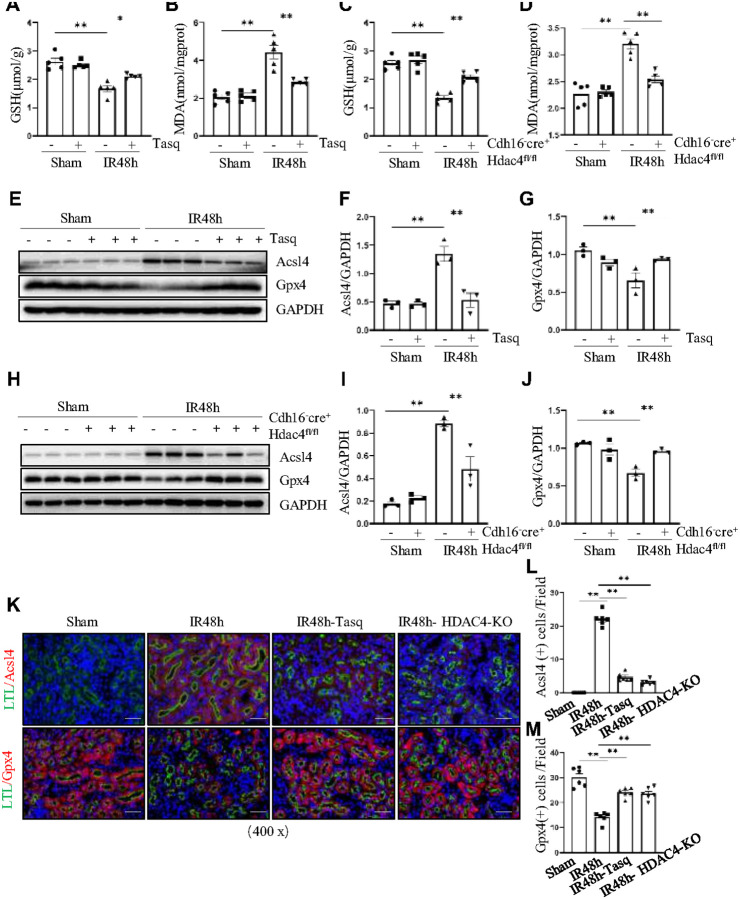
HDAC4 deficiency protects against IR-induced AKI by reducing ferroptosis. The kidney tissues collected at 48 h after sham and IR injury with or without tasquinimod administration in C57/black mice. GSH (A) and MDA (B) levels were measured using specific kits. (E) The kidney tissue lysates were subjected to immunoblot analysis with antibodies against Acsl4, Gpx4 and GAPDH. Expression levels of Acsl4 (F) and Gpx4 (G) were quantified by densitometry and normalized using GAPDH. Cdh16-Cre^−^: HDAC4^fl/fl^ (HDAC4-WT) and Cdh16-Cre^+^: HDAC4^fl/fl^ (HDAC4-KO) mice were sacrificed 48 hours after sham operation or IR. GSH (C) and MDA (D) levels were assessed using specific kits. (H) The kidney tissue lysates were subjected to immunoblot analysis with antibodies against Acsl4, Gpx4 and GAPDH. Expression levels of Acsl4 (I) and Gpx4 (J) were quantified by densitometry and normalized using GAPDH. Values are presented as mean ± SEM of three samples. *p < 0.05; **p < 0.01. (H) After various treatments as indicated, co-staining of Acsl4 and Gpx4 with Lotus tetragonolobus lectin (LTL)-fluorescein. (L) Acsl4 staining shows quantitative data. (M) Gpx4 staining shows quantitative data. Data are presented as mean ± SEM (n = 6 per group). Scale bar =50 μm.

Ferroptosis is a regulated cell death process characterized by iron overload, accumulation of reactive oxygen species (ROS), and lipid peroxidation[33]. To determine whether HDAC4 influences ferroptosis during IR-induced AKI, we first assessed lipid peroxidation and antioxidant capacity. At 48 hours after IR-induced kidney injury, malondialdehyde (MDA), a marker of lipid peroxidation, was significantly increased, while glutathione (GSH) levels were decreased. Treatment with tasquinimod reversed these changes, indicating reduced lipid peroxidation (Fig. 5A, B). In addition, western blot analysis showed that tasquinimod upregulated the ferroptosis-protective marker GPX4 and markedly suppressed ACSL4 expression, which promotes lipid peroxidation (Fig. 5E–G).

Consistently, HDAC4-KO mice exhibited lower MDA levels and partially restored GSH levels compared with WT controls, further confirming HDAC4’s involvement in IR-induced lipid peroxidation (Fig. 5C, D). GPX4 expression, which was impaired in IR-injured WT kidneys, was considerably preserved in HDAC4-KO mice, while ACSL4 expression was significantly reduced (Fig. 5H–J). Immunofluorescence co-localization with the tubular marker Lotus tetragonolobus lectin (LTL) further demonstrated that ACSL4 was abundantly expressed in the cytoplasm of renal tubular cells during IR-induced AKI, whereas its expression was reduced following HDAC4 inhibition or knockout (Fig. 5K, L). Under normal conditions, GPX4 is primarily localized in the cytoplasm of renal tubular cells; however, its expression decreases during ferroptosis following IR. Notably, HDAC4 inhibition or deficiency preserves GPX4 expression under these pathological conditions (Fig. 5K–M).

Together, these findings indicate that HDAC4 promotes lipid peroxidation–mediated ferroptosis in IR-induced AKI, and that pharmacologic inhibition or genetic knockdown of HDAC4 protects the kidney by attenuating ferroptosis.

### HDAC4 inhibition or deficiency protects against IR-induced fibrosis by reducing ferroptosis

**Figure 6:**
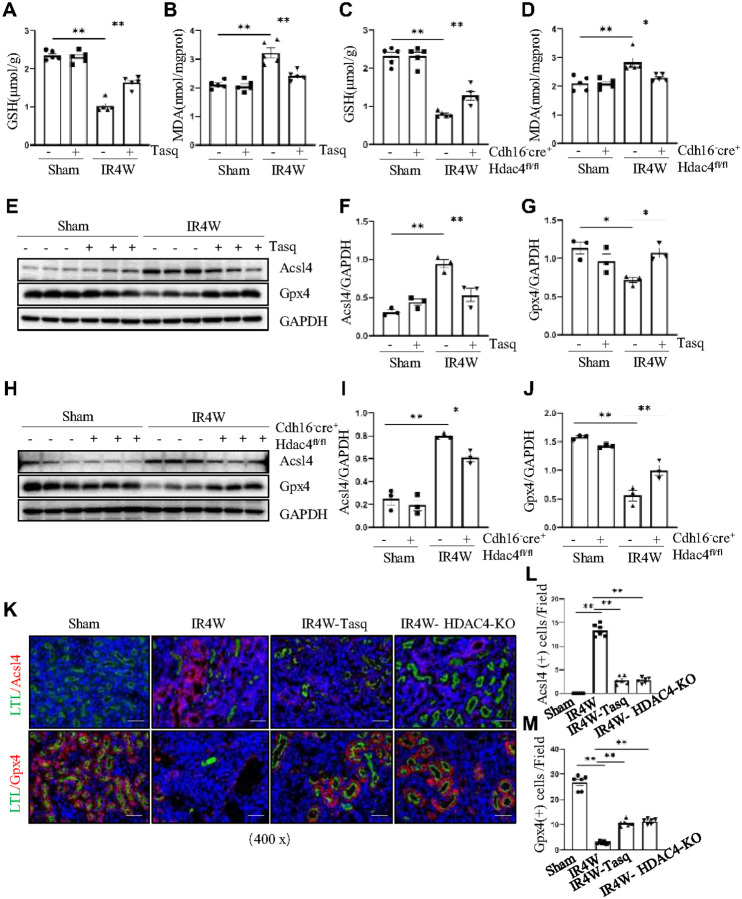
HDAC4 deficiency protects against IR-induced fibrosis by reducing ferroptosis. The kidney tissues collected at 4 weeks after sham and IR injury with or without tasquinimod administration in C57/black mice. GSH (A) and MDA (B) levels were measured using specific kits. (E) The kidney tissue lysates were subjected to immunoblot analysis with antibodies against Acsl4, Gpx4 and GAPDH. Expression levels of Acsl4 (F) and Gpx4 (G) were quantified by densitometry and normalized using GAPDH. Cdh16-Cre^−^: HDAC4^fl/fl^ (HDAC4-WT) and Cdh16-Cre^+^: HDAC4^fl/fl^ (HDAC4-KO) mice were sacrificed 4 weeks after sham operation or IR. GSH (C) and MDA (D) levels were assessed using specific kits. (H) The kidney tissue lysates were subjected to immunoblot analysis with antibodies against Acsl4, Gpx4 and GAPDH. Expression levels of Acsl4 (I) and Gpx4 (J) were quantified by densitometry and normalized using GAPDH. Values are presented as mean ± SEM of three samples. *p < 0.05; **p < 0.01. (H) After various treatments as indicated, co-staining of Acsl4 and Gpx4 with Lotus tetragonolobus lectin (LTL)fluorescein. (L) Acsl4 staining shows quantitative data. (M) Gpx4 staining shows quantitative data. Data are presented as mean ± SEM (n = 6 per group). Scale bar =50 μm.

Single-cell and druggability analyses have identified ferroptosis as a major pathway mediating renal fibrosis[34]. To investigate the role of HDAC4 in ferroptosis during renal fibrosis, we applied either pharmacological inhibition with tasquinimod or conditional HDAC4 knockout as interventions in the unilateral IR model of chronic renal fibrosis. Initial biochemical analyses showed that both tasquinimod treatment and HDAC4 knockdown significantly increased renal GSH levels and reduced MDA levels compared with fibrotic controls (Fig. 6A–D), suggesting that HDAC4 contributes to ferroptosis during renal fibrosis progression.

To further confirm this, we examined key ferroptosis-related proteins. In fibrotic kidneys, HDAC4 and ACSL4 expression were markedly elevated, while the expression of the ferroptosis-protective protein GPX4 was decreased (Fig. 6E–G). These results were reproduced in conditional HDAC4 knockout mice. Ferroptosis was clearly evident in IR-induced renal fibrosis but was substantially ameliorated in HDAC4-KO mice, as indicated by decreased ACSL4 expression and restored GPX4 levels (Fig. 6H–J). Immunofluorescence analyses further supported these findings. In untreated IR-fibrotic kidneys, ACSL4 expression was abundant in the cytoplasm of renal tubular cells, while GPX4 was markedly reduced. In contrast, HDAC4 inhibition or knockout resulted in a significant decrease in ACSL4 fluorescence and a notable increase in GPX4 expression (Fig. 6K–M).

In summary, these results demonstrate that HDAC4 plays a critical role in promoting ferroptosis during renal fibrosis, and that its inhibition or deficiency can effectively mitigate ferroptosis and attenuate IR-induced renal fibrosis.

#### HDAC4 promotes the phosphorylation and nuclear export of Foxo3a, leading to its accumulation in the cytoplasm following IR-induced kidney injury

Foxo3a is a forkhead-type transcription factor known to play a critical role in ferroptosis after IR injury, particularly through its regulation of GPX4[35, 36]. Previous studies have also indicated that the HDAC4–Foxo3a axis is involved in modulating inflammation and oxidative stress[37]. We therefore hypothesized that this axis contributes to IR-induced acute and chronic kidney injury.

**Figure 7:**
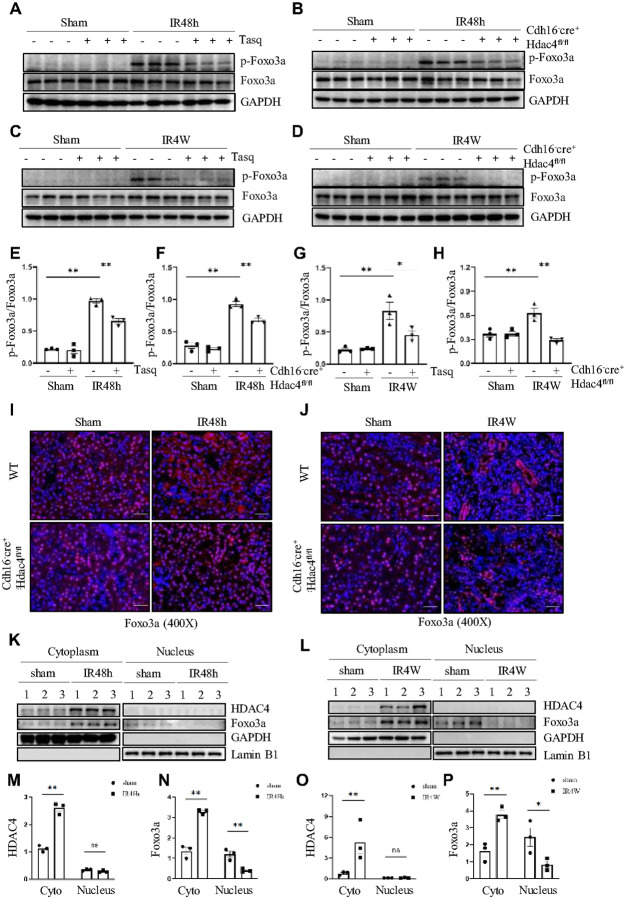
HDAC4 promotes the phosphorylation and nuclear export of Foxo3a, leading to its accumulation in the cytoplasm following IR-induced kidney injury. (A) The kidney tissues collected at 48 h after sham and IR injury with or without tasquinimod administration in C57/black mice. Western blot was performed to detect the protein levels of p-Foxo3a, Foxo3a and GAPDH. (B) Kidney tissues were collected from the injury areas of HDAC4-WT and HDAC4-KO mice at 48 hours after sham operation and ischemia-reperfusion injury. Western blot was performed to detect the protein levels of p-Foxo3a, Foxo3a and GAPDH. (C) The kidney tissues collected at 4 weeks after sham and IR injury with or without tasquinimod administration in C57/black mice. Western blot was performed to detect the protein levels of p-Foxo3a, Foxo3a and GAPDH. (D) Kidney tissues were collected from the injury areas of HDAC4-WT and HDAC4-KO mice at 4 weeks after sham operation and ischemia-reperfusion injury. Western blot was performed to detect the protein levels of p-Foxo3a, Foxo3a and GAPDH. (E-H) Expression levels of p-Foxo3a were quantified by densitometry and normalized using Foxo3a. (I) Kidney tissues were collected from the injury areas of HDAC4-WT and HDAC4-KO mice at 48 hours after sham operation and ischemia-reperfusion injury. Micrographs of Foxo3a immunofluorescence staining were used to assess the nuclear localization of Foxo3a, magnification ×400, Scale bar = 50 μm. (J) The kidney tissues collected at 4 weeks after sham and IR injury in HDAC4-WT and HDAC4-KO mice. Micrographs of Foxo3a immunofluorescence staining were used to assess the nuclear localization of Foxo3a, magnification ×400, Scale bar = 50 μm. (K, L) Kidney samples were collected 48 hours and 4 weeks after IR injury, and then processed using the nuclear and cytoplasmic separation kit. Western blot was performed to detect the protein levels of HDAC4, Foxo3a, GAPDH and Lamin B1. (M-P) The expression levels of HDAC4 and Foxo3a in the cytoplasm were quantitatively determined using the optical density assay method, and normalized with GAPDH as the reference. The expression levels of HDAC4 and Foxo3a in the cell nucleus were quantified and standardized using Lamin B1. Values are presented as mean ± SEM of three samples. *p < 0.05; **p < 0.01, NS means no statistical significance.

To test this, we first examined whether HDAC4 affects Foxo3a expression or activation. Total Foxo3a expression remained unchanged in IR-induced AKI (48 h) and CKD (4 weeks) models (Fig. 7A–D), but western blot analysis revealed increased Foxo3a phosphorylation in both settings (Fig. 7A–H). Immunofluorescence confirmed that this phosphorylation occurred predominantly in the cytoplasm, with elevated levels in renal tubular cells at IR 48 h and IR 4 weeks (Figure S5A–H). Treatment with either tasquinimod or HDAC4 knockdown reduced Foxo3a phosphorylation in both AKI and CKD.

Because Foxo3a’s subcellular localization dictates its transcriptional activity, we next assessed its distribution in the kidney. In healthy tissue, Foxo3a was primarily nuclear; however, after IR injury, it accumulated in the cytoplasm. HDAC4 knockdown reversed this cytoplasmic retention and restored nuclear localization (Fig. 7I, J).

Western blot analysis of cytosolic and nuclear fractions further confirmed that Foxo3a was predominantly nuclear under control conditions, but redistributed to the cytoplasm in both IR-induced AKI and CKD, with concomitant reduction in nuclear localization. This redistribution closely paralleled the accumulation of HDAC4 in the cytoplasm during IR injury (Fig. 7K–P).

Collectively, these findings suggest that HDAC4 regulates Foxo3a subcellular localization by promoting its phosphorylation, thereby restricting its nuclear activity and downstream target gene regulation during IR-induced kidney injury.

#### TGF-β1 treatment promotes a direct interaction between HDAC4 and Foxo3a, resulting in reduced Foxo3a acetylation and increased phosphorylation in cultured renal epithelial cells

**Figure 8:**
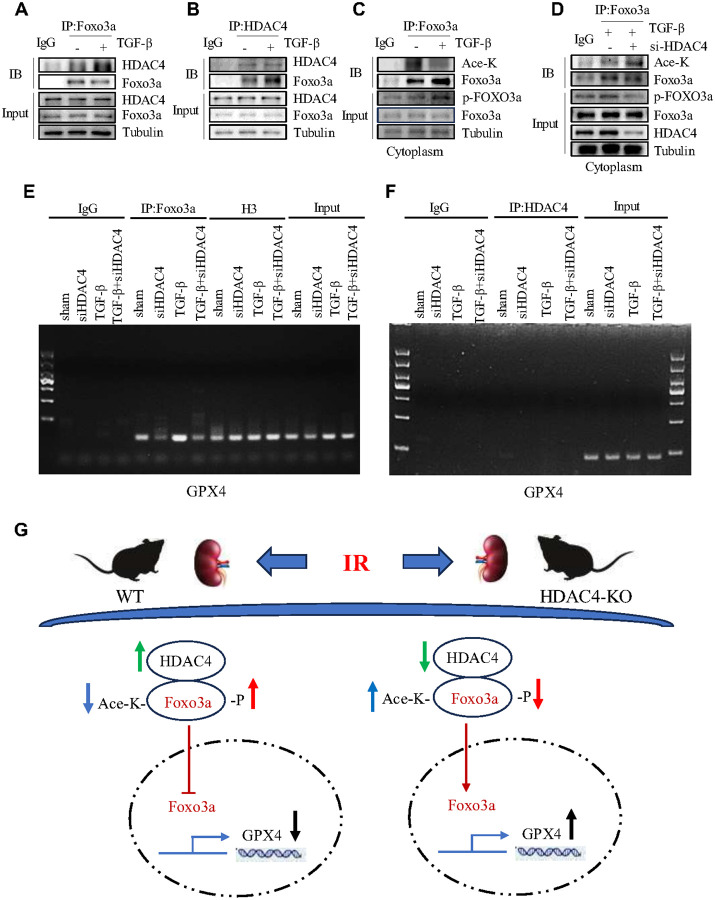
TGF-β1 treatment promotes a direct interaction between HDAC4 and Foxo3a, resulting in reduced Foxo3a acetylation and increased phosphorylation in cultured renal epithelial cells. (A) After TGF-β1 treatment of mTECs, immunoprecipitation with Foxo3a antibody was performed, followed by HDAC4 immunoblotting. (B) After TGF-β1 treatment of mTECs, immunoprecipitation with HDAC4 antibody was performed, followed by Foxo3a immunoblotting. (C) After TGF-β1 treatment of mTECs, immunoprecipitation with Foxo3a antibody was performed, followed by acetyl-lysine and p-Foxo3a immunoblotting. (D) After mTECs were transfected with HDAC4 siRNA or control siRNA and then stimulated with TGF-β1 for 48 hours, immunoprecipitation with Foxo3a antibody was performed, followed by acetyl-lysine and p-Foxo3a immunoblotting detection. (E, F) ChIP assay for Foxo3a and HDAC4 occupancy at the GPX4 Promoter in mTECs with con-siRNA or siHDAC4. After various treatments as indicated, cells for the ChIP assay as described in the materials and methods. Agarose gel electrophoresis of PCR products with IgG samples as negative control. (G) Mechanism of HDAC4 regulation of ferroptosis through Foxo3a. Following IR injury in WT mice, HDAC4 binds to Foxo3a in the cytoplasm and drives the phosphorylation and deacetylation of Foxo3a, inhibiting the nuclear localization of Foxo3a and the transcription of GPX4. In mice with IR injury in conditional knockout models, HDAC4 binding to Foxo3a in the cytoplasm is blocked, Foxo3a undergoes acetylation with concomitant inhibition of phosphorylation, and nuclear localization of Foxo3a is restored and activates GPX4 transcription.

As a deacetylase, HDAC4 may directly interact with Foxo3a and modulate its acetylation. To test this, we performed co-immunoprecipitation (Co-IP) assays. In TGF-β1–stimulated mTECs, HDAC4 was detected in Foxo3a immunoprecipitates but not in IgG controls, confirming their interaction (Fig. 8A). Reciprocal IP with an HDAC4 antibody similarly revealed increased Foxo3a binding under TGF-β1 stimulation (Fig. 8B). Cytoplasmic co-localization of HDAC4 and Foxo3a was further confirmed by fluorescence co-staining, which was lost upon HDAC4 knockdown (Figure S6A), indicating that their interaction occurs primarily under profibrotic conditions.

Given this interaction, we hypothesized that HDAC4 deacetylates Foxo3a in the cytoplasm, thereby regulating its transcriptional activity. Co-IP assays showed that Foxo3a acetylation decreased after TGF-β1 stimulation but was significantly restored by siRNA-mediated HDAC4 knockdown (Fig. 8C, D). Conversely, TGF-β1 increased cytoplasmic Foxo3a phosphorylation, an effect attenuated by HDAC4 knockdown. These results suggest that HDAC4 reciprocally regulates Foxo3a acetylation and phosphorylation, with deacetylation contributing to increased phosphorylation.

Chromatin immunoprecipitation (ChIP) assays demonstrated strong Foxo3a binding to the GPX4 promoter during fibrosis in vitro, which was markedly reduced after HDAC4 knockdown (Fig. 8E). These findings suggest that HDAC4 indirectly suppresses GPX4 transcription by promoting Foxo3a cytoplasmic retention through deacetylation and phosphorylation. Consistently, HDAC4 did not bind the GPX4 promoter under either TGF-β1 stimulation or siRNA-mediated HDAC4 knockdown, excluding the possibility of direct transcriptional regulation by HDAC4 (Fig. 8F).

In summary, during IR-induced AKI and renal fibrosis, elevated HDAC4 promotes Foxo3a phosphorylation and cytoplasmic retention. By binding and deacetylating Foxo3a, HDAC4 impairs its nuclear translocation, reduces GPX4 transcription, and exacerbates ferroptosis. These results identify Foxo3a as a key mediator of the pro-ferroptotic effects of HDAC4 in ischemic kidney injury (Fig. 8G).

## Discussion

AKI is a major risk factor for CKD, with approximately one quarter of AKI cases progressing to CKD. IR injury is the most common driver of this transition, yet the molecular mechanisms linking acute injury to chronic fibrotic remodeling remain incompletely understood. An increasing body of evidence implicates ferroptosis, a regulated form of cell death driven by iron overload, lipid peroxidation, and impaired antioxidant defenses, in both IR-induced injury and renal fibrosis[12, 38, 39]. In this study, we identify histone deacetylase 4 (HDAC4) as a central upstream regulator that connects IR-induced oxidative stress to ferroptosis and subsequent fibrotic progression, thereby bridging the AKI–CKD continuum.

Using IR-induced AKI and CKD mouse models, we found that HDAC4 is rapidly activated following acute ischemia and remains persistently elevated during chronic fibrotic phases. Pharmacologic inhibition with the specific HDAC4 inhibitor Tasquinimod, or conditional genetic deletion of HDAC4 in renal tubules, markedly attenuated tubular injury, preserved renal function, and reduced interstitial fibrosis. These protective effects were associated with a reversal of ferroptotic features, including normalization of GPX4 expression, decreased Acsl4 levels and lipid peroxidation, and restoration of antioxidant capacity. Notably, even in the chronic phase, HDAC4 inhibition corrected the persistent aberrant expression of ferroptosis-related proteins, which was accompanied by a reduction in fibrotic markers such as fibronectin, collagen I, and α-SMA, suggesting that ferroptosis contributes to ongoing fibrotic remodeling.

Mechanistically, we demonstrate that HDAC4 suppresses the expression of GPX4, a key ferroptosis inhibitor, by modulating the post-translational regulation and subcellular localization of the transcription factor Foxo3a. Under physiological conditions, Foxo3a resides predominantly in the nucleus, where it activates antioxidant genes including GPX4[40]. Following IR injury or TGF-β1 stimulation, HDAC4 expression is upregulated and directly binds to Foxo3a in the cytoplasm. This interaction likely promotes Foxo3a deacetylation at yet-unidentified lysine residues, inducing conformational changes that expose phosphorylation sites for upstream kinases, such as AKT and ERK1/2[41]. These kinases have been reported to facilitate Foxo3a phosphorylation at several specific serine/threonine residues, in particular, the phosphorylation of S253 is a crucial residue regulating the nuclear/cytoplasmic shuttling of FoxO3a[41, 42]. Such phosphorylation promotes binding to 14-3-3 proteins, which mask Foxo3a’s nuclear localization signals, thereby preventing its nuclear import and promoting cytoplasmic retention[43]. This dual regulation—deacetylation followed by phosphorylation—renders Foxo3a transcriptionally inactive, suppresses GPX4 expression, and enhances lipid peroxidation, ultimately driving ferroptotic cell death[42]. In support of this model, we found that TGF-β1 exposure reduced Foxo3a acetylation while reciprocally increasing its phosphorylation in renal tubular cells, an effect reversed by HDAC4 inhibition. Similarly, both acute and chronic IR injury increased cytoplasmic Foxo3a phosphorylation in renal tubular cells, which was attenuated by HDAC4 inhibition.

Interestingly, our findings suggest that HDAC4 functions not only as a deacetylase but also as a scaffold protein, bringing Foxo3a into close proximity with kinases such as AKT and MAPKs to facilitate efficient phosphorylation[44, 45, 46]. This scaffold-like role establishes a feed-forward loop in which HDAC4 maintains Foxo3a in an inactive cytoplasmic state, perpetuating oxidative stress and ferroptosis. Disruption of this regulatory complex by HDAC4 inhibition restores Foxo3a acetylation, reduces phosphorylation, promotes nuclear import, and reactivates antioxidant transcriptional programs.

Importantly, the HDAC4–Foxo3a axis appears to operate in both acute and chronic disease contexts. In the fibrotic phase, HDAC4 inhibition not only reactivated Foxo3a and restored GPX4 expression but also attenuated extracellular matrix deposition, indicating that suppression of ferroptosis can limit the profibrotic cascade. This positions ferroptosis not merely as an acute injury mechanism but as a sustained driver of maladaptive repair and fibrosis.

From a therapeutic perspective, our work highlights the HDAC4–Foxo3a–GPX4 pathway as a promising target to break the vicious cycle linking AKI to CKD. While current interventions for AKI are largely supportive, pharmacologic HDAC4 inhibition offers a mechanistically informed approach to preserve antioxidant capacity, reduce ferroptotic injury, and prevent fibrotic remodeling. Given that HDAC4 is druggable and Tasquinimod has been evaluated in clinical trials for other indications[47, 48], there is a translational rationale for repurposing HDAC4 inhibitors in renal injury settings.

However, several questions remain. First, the broader impact of HDAC4 inhibition on other cell death pathways, including apoptosis, necroptosis, and pyroptosis, as well as on inflammatory signaling and non-tubular cell populations, warrants further investigation. Second, given Foxo3a’s context-dependent roles—protective in some settings and deleterious in others—the net effect of its sustained nuclear localization should be evaluated in long-term studies. Third, the specific lysine residue(s) on Foxo3a targeted by HDAC4 for deacetylation, as well as the potential role of this modification in regulating Foxo3a phosphorylation, remain to be identified. Finally, while our work establishes a causal link between HDAC4 activation and ferroptosis in murine models, validation in human AKI–CKD cohorts will be critical for clinical translation.

In summary, we identify HDAC4 as a master regulator of the AKI–CKD transition, acting through coordinated deacetylation and phosphorylation of Foxo3a to repress GPX4, exacerbate lipid peroxidation, and drive ferroptosis. Targeting the HDAC4–ferroptosis axis may offer a novel strategy to mitigate acute injury, preserve renal function, and prevent chronic fibrotic progression.

## Materials and methods

Additional details for all methods are provided in the Supplemental Methods.

### Animal models

All animal experiments in this study were conducted with the approval of the Animal Ethics Committee of Shanghai East Hospital at Shanghai Tongji University. All animals were randomly grouped, and the number of mice used in each group is indicated in figure legends.

### Generation of kidney tubule-specific HDAC4 knockout mice

C57BL/6 male mice were purchased from Shanghai JISJIE Experimental Animal Co., LTD. (Shanghai, China). HDAC4fl/fl and Cdh16-Cre mice (Cdh16-CreER+/−) were purchased from Shanghai Nanfang Model Biotechnology Co., LTD. (Shanghai, China). HDAC4 knockout mice were generated by breeding HDAC4fl/fl with Cdh16-Cre ± mice to obtain Cdh16-Cre+:

HDAC4fl/fl mice (HDAC4-KO) and Cdh16-Cre-: HDAC4fl/fl mice (HDAC4-WT). All animals were housed under standard conditions (12-hour light/dark cycle) with ad libitum access to food and water at the Animal Experimental Center of Tongji University. Mice were used at 8 weeks of age, weighing 20–25 g. All animal experiments were reviewed and approved by the Animal Care and Use Committee of Tongji University.

### Ischemia-reperfusion (IR)-induced AKI and CKD models

AKI was induced by unilateral ischemia-reperfusion. Briefly, the right renal artery and ureter were ligated, the right kidney was removed, and the left renal artery was clamped for 23 minutes at 37°C. In the control group, only the right kidney was removed while the left kidney remained intact. To establish the IR-CKD model, the left renal artery was clamped for 45 minutes at 37°C while preserving the contralateral kidney. In the corresponding sham group, the right renal artery was exposed but not clamped. At least six mice were included per group. For experiments with HDAC4 knockout mice in the IR-AKI model, animals were randomly divided into four groups: (i) Cdh16-Cre^−^: HDAC4fl/fl-Sham group; (ii) Cdh16-Cre^+^: HDAC4fl/fl-Sham group; (iii) Cdh16-Cre^−^: HDAC4fl/fl-IR group; (iv) Cdh16-Cre^+^: HDAC4fl/fl-IR group. For experiments with HDAC4 knockout mice in IR-CKD model, HDAC4 knockout mice were also divided into the same four groups as described above.

For in vivo tasquinimod experiments in AKI, C57BL/6 mice were randomly assigned to four groups: (i) sham-operated group; (ii) tasquinimod-treated sham group; (iii) IR group; and (iv) IR + tasquinimod-treated group. Tasquinimod was dissolved in 5% CMC-Na and administered by gavage at 25 mg/kg/day, starting immediately after reperfusion. Mice receiving only 5% CMC-Na served as vehicle controls. Surgical procedures in knockout mice were identical to those in tasquinimod-treated C57BL/6 mice. All AKI model mice were euthanized with phenobarbital (100 mg/kg) at 48 hours post-surgery, and kidneys and blood were collected for analysis.

For in vivo tasquinimod experiments in IR-CKD, mice were also randomly assigned to four groups: (i) sham-operated group; (ii) tasquinimod-treated sham group; (iii) IR group; and (iv) IR + tasquinimod-treated group. Tasquinimod was administered by gavage at 25 mg/kg/day starting 48 hours after reperfusion and continued daily until day 28; mice receiving only 5% CMC-Na served as vehicle controls. HDAC4 knockout mice were divided into the same four groups as described above, with identical surgical procedures. All CKD model mice were euthanized on day 28, and kidneys and blood were collected for further analysis.

### Cell culture and treatment

Mouse renal proximal tubular epithelial cell lines (mTECs) (Dr. Jeffrey B., Kopp of the National Institutes of Health) were cultured in DMEM with F12 containing 10% fetalbovine serum (FBS) and 0.5% penicillin in an atmosphere of 5% CO2at 37°C. To determine the effect of HDAC4 inhibition on TGF-β1-induced pEMT, siRNA transfection was performed at mTECs densities of 70%. 24 hours after transfection mTECs were incubated with TGF-β1 (5 ng/ml) for 48 hours.

### Western Blot Analysis

Kidney tissue samples were homogenized in RIPA buffer containing phosphatase and protease inhibitors to extract total protein. Protein concentrations were determined using a BCA Protein Assay Kit (Beyotime, Shanghai, China). Equal amounts of protein were separated by sodium dodecyl sulfate–polyacrylamide gel electrophoresis (SDS-PAGE) and then transferred onto PVDF membranes (Millipore, Bedford, MA, USA). Membranes were blocked with 5% nonfat milk for 1 hour at room temperature, incubated with the appropriate primary antibodies overnight at 4°C, and then incubated with the corresponding secondary antibodies for 1 hour at room temperature. Protein bands were visualized and quantified using ImageJ software (National Institutes of Health, Bethesda, MD, USA).

### Co-immunoprecipitation (Co-IP) Assay

Total protein was extracted from mTECs treated with or without TGF-β1 stimulation for 48 hours using cell lysis buffer. To assess the binding of HDAC4 to Foxo3a and the acetylation level of cytoplasmic Foxo3a, co-immunoprecipitation was performed. After 24 hours of HDAC4 transfection or control treatment, mTECs were stimulated with TGF-β1 for 48 hours, and cytoplasmic proteins were collected. The interaction between Foxo3a and acetyl-lysine (ace-K) was examined by immunoprecipitation using Protein A/G Magnetic Beads (HY-K0202) following the manufacturer’s protocol. Beads were washed five times with PBST (0.05% Tween-20), and immunoprecipitated complexes were eluted with 1× SDS loading buffer by heating in a metal bath at 100°C.

### Immunofluorescence

Immunofluorescence staining of kidney tissues was performed as described in our previous study[49]. Briefly, kidney sections were incubated with primary antibodies against Ngal (sc-515876), α-SMA (ab5694), HDAC4 (sc-46672), p-H3 (NB21–1091), Acsl4 (A20414), Foxo3a (A0102), phospho-Foxo3a (Ser253; AP0684), and GPX4 (ab125066), as well as Lotus tetragonolobus lectin (LTL)–fluorescein (#L32480, Thermo Fisher). After primary incubation, sections were incubated with appropriate fluorescence-labeled secondary antibodies. Images were acquired randomly using an Olympus BX53 microscope at ×200 magnification, and fluorescence intensity was quantified with ImageJ software (NIH, USA).

For cell staining, mTECs with different treatments were cultured on coverslips, fixed with 4% paraformaldehyde, and blocked at room temperature for 1 hour in blocking buffer (5% sheep serum in PBS). Cells were then incubated overnight at 4°C with the primary antibody, washed three times with PBS, and incubated with the appropriate fluorescence-labeled secondary antibody for 1 hour at 37°C. Nuclei were counterstained with DAPI. Immunofluorescence images were acquired using a ZEISS digital fluorescence microscope.

### Pathological Assessment

Kidney tissues were fixed in formalin, embedded in paraffin, and sectioned at a thickness of approximately 3–5 μm. Periodic acid–Schiff (PAS) staining and Masson’s trichrome staining were performed according to standard protocols to evaluate renal injury and fibrosis, respectively. The severity of tubular injury and the extent of renal fibrosis were assessed by light microscopy in at least 10 randomly selected fields per kidney section. Tubular injury was scored as previously described: 0, none; 1, < 10%; 2, 10–25%; 3, 26–50%; 4, 51–75%; and 5, ≥ 75%[50].

### Renal Function Analysis

Serum was prepared by centrifuging whole blood at 1,500 rpm for 15 minutes at 4°C. Serum creatinine (Scr) levels were measured using a creatinine detection kit, and serum blood urea nitrogen (BUN) levels were determined using a BUN assay kit, following the manufacturers’ instructions.

### Chromatin Immunoprecipitation Assay (ChIP)

ChIP assays were performed using the SimpleChIP Plus Enzymatic Chromatin IP Kit (Magnetic Beads) (#9005, Cell Signaling Technology) following the manufacturer’s instructions with minor modifications. Briefly, minced kidney tissues were cross-linked in phosphate-buffered saline (PBS) containing 1.5% formaldehyde and 1× protease inhibitor cocktail (PIC) at room temperature for 20 minutes. Chromatin was enzymatically digested with Micrococcal Nuclease and further sonicated to yield soluble chromatin fragments of 200–500 base pairs. A 2% aliquot of chromatin was saved as input control. Immunoprecipitations were carried out overnight at 4°C using antibodies against HDAC4 (sc-46672, Santa Cruz), Foxo3a (A0102, ABclonal), Histone H3 (#4620, Cell Signaling Technology), or IgG (#2729, Cell Signaling Technology) as a negative control. Protein-DNA complexes were captured with ChIP-Grade Protein G Magnetic Beads (#9006), eluted, and reverse cross-linked. After protease K treatment, DNA was purified using DNA purification columns and analyzed by PCR with primers targeting the GPX4 promoter region:

Forward primer: GGGCTCTTTAAGGGGATGAC
Reverse primer: GTTATCCGCGAGGTTGCCTG

Quantitative PCR data were normalized to 1% input chromatin DNA and expressed as fold enrichment relative to IgG controls. Input control represents non-immunoprecipitated total chromatin DNA.

### Statistical Analyses

Data are presented as mean ± the standard error of the mean (SEM). Intergroup comparisons were performed using one-way ANOVA. Statistical significance was indicated in the figures, with *p* < 0.05 considered statistically significant.

## Supplementary Material

This is a list of supplementary files associated with this preprint. Click to download.

- Supplementaldata.pdf

## Data Availability

The data that support the findings of this study are available from the corresponding author upon reasonable request.
